# Situational analysis of antibiotic use and resistance in Ghana: policy and regulation

**DOI:** 10.1186/s12889-017-4910-7

**Published:** 2017-11-23

**Authors:** Saviour Kwame Yevutsey, Kwame Ohene Buabeng, Moses Aikins, Berko Panyin Anto, Richard B. Biritwum, Niels Frimodt-Møller, Martha Gyansa-Lutterodt

**Affiliations:** 1 Faculty of Pharmacy and Pharmaceutical Sciences, College of Health Sciences, Department of Pharmacy Practice, Kwame Nkrumah University of Sciences and Technology, Kumasi, Ghana; 2 School of Public Health, College of Health Sciences, University of Ghana, Legon, Ghana; 3 Department of Community Health, University of Ghana, Legon, Ghana; 4 Department of Clinical Microbiology, Unit 9301, Rigshospitalet, Blegdamsvej 9, DK-2100 Copenhagen, Denmark; 5 Director of Pharmaceutical Services, Ministry of Health, Accra, Ghana

**Keywords:** Antibiotics, Regulation, Policy, Situational analysis, Ghana

## Abstract

**Background:**

Antibiotics have played an essential role in decreasing morbidity and mortality from infectious diseases. However, indiscriminate use and unrestricted access is contributing to the emergence of bacterial resistance. This paper reports on a situational analysis of antimicrobial use and resistance in Ghana, with focus on policy and regulation.

**Methods:**

Relevant policy documents, reports, regulations and enactments were reviewed. PubMed and Google search engines were used to extract relevant published papers. Websites of stakeholders such as Ministry of Health (MOH) and its agencies were also reviewed. An interview guide was used to elicit responses from selected officials from these sectors.

**Results:**

Laws and guidelines to control the use of antimicrobials in humans were available but not for animals. There was no National Antimicrobial Policy (NAP). A health practice regulatory law mandates Physicians, Physician Assistants, Midwives and trained Nurses to prescribe antimicrobials. However, antibiotics are widely prescribed and dispensed by unauthorised persons, suggesting weak enforcement of the laws. Antibiotics were also supplied to and from unapproved medicine outlets. The Standard Treatment Guidelines (STG), Essential Medicines List (EML) and the National Health Insurance Scheme Medicines List (NHISML) provide restrictions regarding levels of prescribing of antimicrobials. However, existing guidelines on antibiotic use are mostly not adhered to. The use of Automatic Stop Orders to avoid wastage in the hospitals is also not practiced. Data on use of antibiotics for individuals are not readily available in most facilities. Again, there are no standards or guidelines on veterinary use of antibiotics. Surveillance systems for consumption of antibiotics and resistance monitoring were not in place in most health facilities. However, there is an ongoing national action to create awareness on bacteria resistance, strengthening knowledge through research and surveillance and development of NAP in line with global action plan on antimicrobial resistance.

**Conclusion:**

Absence of national antimicrobial policy, weak regulatory environment and non-adherence to practice standards may have contributed to increased and unregulated access to antimicrobials in Ghana, a catalyst for development and spread of antimicrobial resistance.

**Electronic supplementary material:**

The online version of this article (10.1186/s12889-017-4910-7) contains supplementary material, which is available to authorized users.

## Background

Antimicrobial drugs have played an essential role in decreasing morbidity and mortality due to infectious diseases since their introduction in the 1940’s. Infectious diseases account for more than 11 million deaths worldwide in 2000 [1]. Resistance though a natural phenomenon of microbes, has seen an increase in prevalence and spread over the years largely due to inappropriate use of antibiotics both in health facilities and the community [1, 2]. Antimicrobial resistance (AMR) is not limited to developed countries. Emerging economies are also experiencing accelerating rates of AMR, including the spread of new multi-drug resistance strains of pathogenic microbes [3].

The economic burden of antibiotics use and antibiotic resistance on individuals in society, health systems and governments cannot be over-simplified. Kim et al., found that patients infected with antimicrobial-resistant organisms have higher costs compared to patients with infections due to antimicrobial-susceptible organisms [4]. Cost of treatment is also high when patients are infected with resistant organisms compared to patient with no infection [5]. In 2007, resistant bacterial infections accounted for about 2.5 million extra hospital days. It also resulted in societal cost of 1.5 billion Euros per year [6]. The mean cost per patient for the hospitals is estimated to range from 51,252 to 84,436 USD for Methicillin-resistance *Staphylococcus aureus* (MRSA) infections compared with 30,158–59,245 USD for Methicillin-sensitive *Staphylococcus aureus* (SSA) [7]. Another major consequence of resistant bacterial infections is the use of extended spectrum and most often expensive antibiotics [8].

In Ghana specifically, there is a dearth of evidence on the cost, use of, as well as resistance to antibiotics. Antimicrobial resistance is a major public health concern in Ghana as it is difficult to therapeutically manage infections from resistant strains of bacteria and could thus spread rapidly within the population into endemic and epidemic proportions.

Several studies have demonstrated high prevalence of resistance to commonly used antibiotics such as tetracycline (82%), ampicillin (76%), chloramphenicol (75%) and cotrimoxazole (73%) [9, 10]. Penicillin is gradually losing its effectiveness against *Streptococcus pneumoniae* and *Neisseria meningitidis* in Ghana [11–13].

Poor prescription and dispensing practices, poor adherence to antibiotic treatment by patients, and the supply and use of substandard antibiotics for treatment of microbial infections are among the determinants of AMR. It has been established that higher rate of oral antimicrobial treatment corresponds to elevation in resistance [14]. In the United State, 22.7 million kg of antibiotic per annum are prescribed for humans [15].

The inappropriate use of antimicrobial drugs in animal husbandry usually results in exposing microorganisms to low concentrations of the agents over a long period of time and emergence of resistant strains that are then transmitted to humans [16, 17]. In humans, inappropriate use of antibiotics for conditions such as common cold, availability of antibiotics over the counter and their sub-optimal use provide opportunity to kill susceptible bacteria and allow resistant bacteria to survive and multiply [18, 19].

This paper provides situational analysis of antibiotic use and resistance in humans and animals in Ghana with focus on policy and regulation.

## Methods

A situational analysis was conducted using multiple data collection methods. The assessment was carried out in the context of policy and regulatory environment of antibiotic use and resistance in Ghana. The Ministry of Health develops health policies which are implemented by several implementing agencies. The Ghana National Drug Policy guides the pharmaceutical sector and the use of medicines in the country [20].

Relevant policy documents were reviewed including enactments regulating prescribing, dispensing and use of medicines. Web literature search was conducted for relevant published papers using PubMed and google search engines. The search terms used include “antimicrobial policy Ghana”, “antimicrobial use Ghana”, “antimicrobial resistance Ghana”, “prescribing practices antimicrobial Ghana”, “community antimicrobial use Ghana”, “antimicrobial regulation Ghana”. The terms “antibiotic” and “antibacterial” were used to replace the term ‘antimicrobial’ in conducting the search. Websites of relevant stakeholders such as Ministry of Health (MOH), National Health Insurance Authority (NHIA), Pharmacy Council (PC), Ghana Health Service (GHS), Ministry of Foods and Agriculture (MOFA), Antibiotic Drug use, Monitoring and Evaluation of Resistance (ADMER) Project, Ghana National Drug Programme (GNDP), Food and Drug Authority (FDA) in the field of antibiotic use and regulation were searched for related reports and policy documents. Unpublished literature was also accessed at the Kwame Nkrumah University of Science and Technology (KNUST) seminars.

### Review of documents

The areas of document review included; existing structures relevant to the broad area of antibiotic use and resistance; legislative framework/regulatory environment; policies and guidelines/administrative and management arrangement; functions to be achieved by the structures and frameworks indicated; extent of the problem of antimicrobial resistance; underlying determinants contributing to the problem of abuse and resistance of antibiotics; challenges in existing attempts to solve the problem. The reviewed policy documents are provided as “Additional file 1”.

### Interviews with key informants

Interviews were conducted with four purposively selected respondents using an interview guide. Respondents included senior officials from the Ministry of Food and Agriculture (MOFA) specifically in the Veterinary Department, Ministry of Fisheries and Aquaculture, Ministry of Health and the academia (KNUST). Through face to face interviews which lasted on average 20mins, views of respondents pertaining to availability of policies and guidelines on antimicrobial use and resistance in humans and animals, surveillance system for consumption of antimicrobial and antimicrobial resistance, treatment guidelines, laws governing prescribing of antimicrobials and policy on limits of antimicrobials residues in animal products were collected. Further, interviews focused on clarifying, as well as further exploration of the issues identified from the review of documents were conducted.

Data were analysed using deductive content thematic analysis. The themes that were derived from the review of documents guided the analysis. Findings from the various approaches were put together under relevant themes of the study.

## Results

### Health system and service delivery

Health services are delivered through a three tier system namely primary, secondary and tertiary and the use of antimicrobials are tied to these levels of care. Ministry of Health develops Essential Medicines List (EML), Standard Treatment Guidelines (STG) and the National Drug Policy to be implemented by the implementing agencies [21]. The FDA of Ghana is responsible for regulation of all medicines including antimicrobials.

### Policy and regulatory environment of antibiotic use and resistance

Table 1 shows the key policy and regulation issues of antimicrobial use and resistance from the interviews and document reviews.

**Table 1:** Key issues on policy and regulation of antibiotics in human in Ghana, 2015

Number	Issues
1	There is no National Antimicrobial Policy
2	There is easy access to restricted antibiotics in unlicensed sources and premises
3	Antibiotics are prescribed and dispensed by unapproved health professionals
4	There is no system for surveillance of consumption of antibiotics in the country.
5	There is no system for antimicrobial resistance surveillance for both humans and animals

Though there is National Drug Policy regulating the use of all medicines in Ghana, the provisions in the policy do not provide for control of antibiotic resistance and use. There is no separate policy to address the issues of resistance and antibiotic use under the One Health Concept as proposed by the WHO. The effect of this gap is the easy access to antibiotics as is observed in many countries in Africa, where antibiotics are readily available on demand from hospitals, community based retail pharmacies, roadside stalls, retail Licensed Over the Counter Medicine suppliers (OCMS) and hawkers [22, 23].

According to the Ghana National Drug Policy, prescribing of medicines shall be in accordance with the Health Professions Regulatory Body Act, (Act 857), 2013. Prescribers who are of good standing with their respective professional associations are legally permitted to prescribe medicines. Persons eligible to prescribe antibiotics and other essential medicines are the medical doctors, physician assistants, midwives and nurses trained in prescribing [24].

The FDA is responsible for drug regulation in Ghana [25]. The authority classified all medicines in to three categories namely Over-The-Counter (OTC) medicines, Pharmacy Medicines (PM), and Prescription Only Medicines (POM). Antibiotics used in Ghana are classified by the FDA in to three categories as shown in Table 2.

**Table 2:** Classification of Antibiotics by the FDA of Ghana in 2015

Over the Counter Antibiotic	Pharmacy Medicines (Antibiotics)	Prescription Only Medicines
Cotrimoxazole suspension	Amoxicillin	All registered Antibiotics
	Flucloxacillin	
	Norfloxacin + Tinidazole	
	Ciprofloxacin	
	Doxycycline	
	Tetracycline	
	Erythromycin	
	Ampicillin	

Pharmacy medicines do not require medical practitioner’s prescription before dispensing, but may only be supplied on the recommendation of a pharmacist on professional judgement and maintain proper records on the transactions [26]. Except for eye ointments containing 1% Tetracycline or Oxy-tetracycline which are for use in Trachoma and Chloramphenicol eye preparations; Amoxycillin, Flucloxacillin and Norfloxacin + Tinidazole oral preparations; Ciprofloxacin, Doxycycline, Tetracycline and Erythromycin for the treatment of sexually transmitted infections, all antibiotics are classified as POM and therefore requires that they must be prescribed by legally permitted clinicians only [25, 26]. Evidence available suggests that antibiotics are prescribed and dispensed by persons not mandated by the Regulatory Act especially in the rural areas of the country [27, 28]. This creates avenue for inappropriate use of antibiotics and the tendency of creating opportunity for antibiotic resistance in the community.

In enhancing the appropriate use of medicines including antibiotics, the MOH has developed STG that guide health professionals in the choice of medicines for treating diseases. Based on the recommendations from the STG, the Ghana EML was also developed. It serves as basis for public procurement of medicines. The National Health Insurance Scheme Medicines List (NHISML) which is used under the health insurance scheme to reimburse drug claims from healthcare providers is also developed from the STG.

The adherence to the STG in the management of infectious diseases was found to be problematic resulting in high usage of antibiotics at the facility level [29]. Prescribers were found to be prescribing antibiotics to all non-pneumonia Acute Respiratory Infections (ARI). Over 40% of non-bacteria diarrhoea in children received antibiotics not in line with the STG. More antibiotics are prescribed at the lower levels of the heath care settings compared to the referral centres [29, 30]. Antibiotics are inappropriately prescribed mostly in the rural and peri-urban areas in Ghana under the National Health Insurance Scheme [31].

### Use of antibiotics in humans

**Fig. 1:**
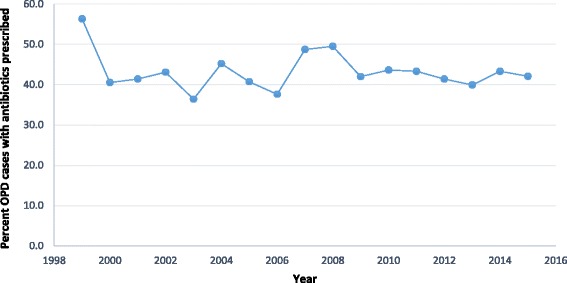
Percentage of OPD (out-patient department) cases that received antibiotics from 1999 to 2015 in Ghana. This was extracted from reports on monitoring of prescribing habits of health care providers across the country

The Ministry of Health (MOH) in 1998 adopted the Rational Use of Medicines (RUM) concept with the aim of improving the use of medicines in Ghana. The Ghana National Drug policy stipulates that there should be routine monitoring of RUM [20]. In order to monitor the improvement or otherwise of antibiotic use, the WHO core indicator on antibiotic use at the out-patient department (OPD) was introduced; namely percentage of encounters with an antibiotic prescribed [32]. The results of this monitoring are shown in Fig. 1.

### Restriction on antibiotic use

National policy documents that provide restrictions on the use of antibiotics across the country are listed in Table 3.

**Table 3:** Policy documents providing restriction on antibiotic use in Ghana, 2015

Number	Policy document
1	Standard Treatment Guidelines of Ghana
2	Essential Medicines List
3	National Health Insurance Scheme Medicines List

National therapeutic guidelines for practitioners of orthodox medicines are to be revised and distributed to all registered orthodox health care practitioners to serve as a guide in the treatment of most ailments in the country [20]. These documents provide some level of restrictions on prescribers and dispensers on the choice and use of antibiotics for selected bacterial infections presenting at the various levels of health facilities in the country. The EML also provides restrictions on the level at which the selected antibiotics are to be used across the spectrum of health facilities [20]. The NHISML with which medicines are reimbursed provides restrictions on prescribing antibiotics by the levels of health care.

Out of 548 medicines of different strengths and dosage forms on the NHISML, 72 (13.1%) are antibiotics. Sixteen items representing 22% are restricted to be prescribed by midwives [33]. They include penicillins, quinolones, and the macrolides (erythromycin). Physician/Medical Assistants who practice at the Health Centre level and above, could only prescribe 46 out of the 72 antibiotics and the facility will be reimbursed by National Health Insurance Authority (NHIA). The NHISML and the EML restrict Medical Assistants from prescribing Cephalosporins [34]. Chloramphenicol capsules that have not been listed in the STG and EML of 2010 because of bacterial resistance have been added to the NHISML.

The use of other forms of restrictions to improve on use of antibiotics such as Automatic Stop Orders in order to avoid wastage in the hospitals, are not complied with [33]. Antibiotics are classified as class A medicines and their use is to be controlled using the Restricted Medicines Records Book (RMRB) as recommended by Health Professions Regulatory Body Act [24], however, this is not adhered to. Therefore, data on the use of antibiotics for individuals are not readily available at the health facilities [22].

### Use of antibiotics in animals

There were several policy and system gaps observed from the interviews and document reviews in antimicrobials use and resistance in Ghana as indicated in Table 4.

**Table 4:** Key issues identified on policy, regulation and antibiotics use in animals in Ghana, 2015

Number	Issues
1	There is legal framework designed to control distribution and use of antibiotics in humans. Such a design could not be said for the control, distribution and use of antibiotics in veterinary and aquaculture practice.
2	Lack of records on the use of antibiotics in veterinary and aquaculture.
3	There is distribution of antibiotics from unapproved sources.
4	Absence of policy directives on veterinary use of antibiotics with focus on Acceptable Daily Intake (ADIs) of antibiotics, Maximum Residue Limits (MRLs) and withdrawal periods
5	There is no national surveillance system on antimicrobial use and resistance

The minimum allowable weaning period before slaughter and processing of animals have not been established. Personal communication with a senior officer of the Ministry of Agriculture, veterinary department (2014) suggests that policy provisions on Acceptable Daily Intake (ADIs) of antibiotics, Maximum Residue Limits (MRLs) and withdrawal periods are yet to be established for antibiotic use in animals.

## Discussion

Our study has shown gaps in the policies, weak adherence to regulations and guidelines, weak monitoring and contradictions in policy directives as major issues likely to contribute to anti-microbial resistance.

### Policy gaps

The absence of a national policy on antibiotics use and containment of AMR provide disturbing scenarios for the fight against AMR in the country. There were no shared efforts among users of antibiotics by policy directions in addressing the issues of AMR under the one health concept. Ghana was one of the several countries without national action plan on the containment of AMR [35].

Antibiotic resistance has not been a priority for the veterinary services in Ghana. The absence of national standards for antibiotic residue in veterinary and aquaculture products and surveillance testing for antibiotic residue is manifest in the studies conducted in Ghana. Antimicrobial residues were detected in meat and poultry products in the country [36]. The uncontrolled use of antibiotics in animals may lead to unacceptable levels of antibiotic residues in the meat products for human consumption. Consumption of these products may expose consumers to sub-optimal doses of these antibiotics which may lead to selective pressure of resistant strains. It may lead to breeding more resistant strains of bacteria that could be transmitted from animals to humans [37]. Since there are no formalised systems to control the use of antimicrobial agents in veterinary and aquaculture, the consumption data of these drugs are not available. The antimicrobial consumption surveillance drive of the World Health Organization may have setback if these systems are not in place.

### Adherence to regulations, guidelines, and policies

Enforcement of laws regulating drug distribution and use in the country is vital in achieving the target of the sector [24]. The easy access to restricted drugs depicted in the results will encourage inappropriate use of antibiotics. This phenomenon has the tendency to encourage overuse of antibiotics and the development of resistant bacterial strains.

The situation where antibiotics are prescribed and dispensed by unauthorised persons in the health system is at variance to the Health Professions Regulation Act, 857 (2013). This unauthorised practice may create avenue for inappropriate use of antibiotics and the tendency of creating opportunity for antibiotic resistance in the community. This may have negative treatment outcomes and high health care cost to the patients and to the state [38, 39].

### Weak monitoring

Absence of national surveillance system for monitoring AMR has serious effects on knowing the pattern of resistance to commonly used antibiotics in the health system. Selection of antibiotics for the treatment of infectious diseases may therefore not be informed by AMR surveillance data. Ghana may be losing resources in the treatment of resistant cases at the service delivery points. Contribution of the country to the knowledge of global burden of antibiotics resistance may not be known and therefore losing the fight against AMR in the global action.

Consumption data on antibiotics play critical role in antibiotic consumption surveillance at the national and the facility levels. Lack of such data is a serious setback to antibiotic consumption monitoring and relating it to AMR as there is correlation between use of antibiotics and resistance development [40]. Ghana may be losing the opportunity to contribute to the international picture of antibiotic consumption monitoring envisaged in the Global Action Plan on AMR [41].

As a target, at the out-patient-department (OPD), not more than 30% of encounters are to be prescribed one or more antibiotics [42]. Since the inception of the monitoring of this indicator, the country has not been able to achieve the set target. The drop from 56.3% in 1999 to 42.1% was as result of continuous training of health care providers through the Drugs and Therapeutics Committee platform in the facilities as reported in unpublished annual reports of the Regional Health Administrations. Additional research needs to be conducted to determine whether the approximately 40% OPD visits receiving antibiotics at the hospital is legitimate. The ease of accessing antibiotics without prescription may contribute to misuse and hence AMR development [43, 44]. This practice makes the fight against AMR locally and internationally a challenging one.

### Contradictions in the policy directives

Several studies revealed high resistance to chloramphenicol necessitating its removal from the STG and the EML [45–47]. The removal of Chloramphenicol from the STG provides restriction on its use in the health facilities. The NHIS however, listed it to be prescribed for the treatment of infections. This disagreement between two state policy guidelines creates opportunity for non-compliance. The continuous use of this drug may lead to increased health care cost and adverse effects for the patients, society and the state.

## Conclusion

Absence of National Antimicrobial Policy for both humans and animals, coupled with weak enforcement of existing regulations and non-adherence to practice standards could make it easy for people to access antimicrobial agents and use it indiscriminately. This is known to be a major determinant for antimicrobial resistance and its spread in the health system. National effort initiated to address this challenge must be sustained for public health good.

## Additional file

Additional file 1: List of reviewed documents. (DOCX 11 kb)

## Abbreviation

AMR: Antimicrobial resistance
EML: Essential Medicines List
FDA: Food and Drug Authority
GNDP: Ghana National Drug Programme
MOH: Ministry of Health
NAP: National Antibiotic Policy
NHIA: National Health Insurance Authority
NHISML: National Health Insurance Scheme Medicines List
STG: Standard Treatment Guidelines
WHO: World Health Organization
