# Supplementary material for: Situational analysis of antibiotic use and resistance in Ghana: policy and regulation
Source: BMC Public Health. 2017 Nov 23;17:896. doi: 10.1186/s12889-017-4910-7 (PMC5701378; doi:10.1186/s12889-017-4910-7)
Supplement: Additional file 1: — List of reviewed documents. (DOCX 11 kb) [file 12889_2017_4910_MOESM1_ESM.docx]

**Additional file 1**

**List of reviewed documents**

National Health Policy, 2007

Ghana National Drug Policy, 2^nd^ Edition

Standard Treatment Guidelines, 2010

Essential Medicines List of Ghana, 2010

National Health Insurance Medicines List, 2012

Medicine Classification List of Food and Drugs Authority of Ghana, 2015

Health Professions Regulatory Act, 857, 2013
